# Collaboration between specialties for respiratory allergies in the International Classification of Diseases (ICD)-11

**DOI:** 10.1186/s12931-017-0513-1

**Published:** 2017-02-10

**Authors:** Luciana Kase Tanno, Moises Calderon, Jeffrey F. Linzer, Robert J.G. Chalmers, Pascal Demoly, on behalf of the Joint Allergy Academies

**Affiliations:** 1 Hospital Sírio Libanês, São Paulo, Brazil; 2 Division of Allergy, Department of Pulmonology, Hôpital Arnaud de Villeneuve, University Hospital of Montpellier, 371, av. du Doyen Gaston Giraud - 34295, Montpellier Cedex, 5 France; 3 Sorbonne Université, UPMC Paris 06, UMR-S 1136, IPLESP, Equipe EPAR, 75013, Paris, France; 4 Section of Allergy and Clinical Immunology, Imperial College London, National Heart and Lung Institute, Royal Brompton Hospital, London, United Kingdom; 5 Division of Pediatric Emergency Medicine, Children’s Healthcare of Atlanta at Egleston, Emory University School of Medicine, Atlanta, GA USA; 6 Co-Chair and Managing Editor, Dermatology Topic Advisory Group, ICD-11 Revision Steering Group, University of Manchester, Manchester, UK

**Keywords:** Allergy, Asthma, Hypersensitivity, Rhinitis, Respiratory allergies, International Classification of Diseases (ICD), World Health Organization (WHO)

## Abstract

**Background:**

The International Classification of Diseases (ICD) has been grouping the allergic and hypersensitivity disorders involving the respiratory tract under topographic distribution, regardless of the underlying mechanisms, triggers or concepts currently in use for allergic and hypersensitivity conditions. In order to strengthen awareness and deliberate the creation of the new “Allergic or hypersensitivity disorders involving the respiratory tract” section of the ICD-11, we here propose make the building process public.

**Methods:**

The new frame has been constructed to cover the gaps previously identified and was based on consensus academic reports and ICD-11 principles. Constant and bilateral discussion was kept with relevant groups representing specialties and resulted in proposals submission into the ICD-11 online platform.

**Results:**

The “Allergic or hypersensitivity disorders involving the respiratory tract” section covers 64 entities distributed across five main categories. All the 79 proposals submitted resulted from an intensive collaboration of the Allergy working group, relevant Expert working groups and the WHO ICD governance.

**Conclusion:**

The establishment of the ICD-11 “Allergic or hypersensitivity disorders involving the respiratory tract” section will allow the dissemination of the updated concepts to be used in clinical practice by many different specialties and health professionals.

## Background

### Allergic and hypersensitivity disorders involving the respiratory tract

The World Health Organization (WHO) defines non-communicable chronic respiratory diseases (CRDs) as conditions of the airways and other structures of the lung, such as asthma, chronic obstructive pulmonary diseases (COPD), occupational lung diseases, cystic fibrosis and pulmonary hypertension. Changes in life-style and in exposure to environmental pollutants and allergens have increased both the prevalence and the severity of CRDs in different age groups, with increased impact on quality of life and healthcare costs, as has been shown particularly for asthma.

Over the last few decades, the world has been facing an asthma and allergy epidemic, becoming a serious public health problem and a prominent cause of disability and mortality worldwide. The WHO estimates that about 235 million people currently suffer from asthma [1]. Although most common in childhood, asthma can persist into adulthood and the prevalence can vary widely across the age groups [1].

Though asthma mortality rates are higher in lower and lower-middle income countries, it has been accepted as a major public health problem in all countries. Among the European Union (EU) member states, asthma accounted for an average of 53 hospital admissions per 100,000 inhabitants in 2009 [2]. The annual direct and indirect costs in European countries due to asthma are estimated at €22,2 billion and €16,4 billion respectively per year. Data on the value of disability-adjusted life-years lost due to asthma obtained from WHO Health Statistics 2011 and the Global Burden of Disease study indicate an estimated monetized total cost of €43.2 billion [3, 4].

Allergic and hypersensitivity disorders involving the respiratory tract comprise a range of different clinical presentations including asthma, rhinitis, and pneumonitis among others. They can manifest at any age and in differing degrees of severity, frequently giving rise to a significant impact on the quality of life of patients and their families. They are likely to be encountered at some stage by every health professional.

Many international initiatives have been launched to prevent and decrease the negative outcome of these conditions, which are considered a global health problem [5, 6]. However, international classification and coding systems such as the International Classification of Diseases (ICD) have grouped them under topographic distribution, regardless of the underlying mechanisms and triggers. This has led to the framework of both the 10th and draft 11th revisions of ICD (ICD-10 2010 version and ICD-11 beta draft as at May 2014 respectively) to be deficient in capturing the concepts currently in use for allergic and hypersensitivity conditions [7]. These findings highlight the need for the major respiratory societies to be involved in updating the representation of allergic and hypersensitivity disorders involving the respiratory tract in the ongoing ICD-11 revision.

### Rationale for updating allergic and hypersensitivity disorders involving the respiratory tract in the ICD-11 framework

#### The allergy specialty scope

Allergy and clinical immunology, while perceived as a secondary discipline in some medical communities, has been increasingly recognized around the world as a key specialty in its own right [8, 9]. Unlike many other specialties, which focus mostly on specific organ systems, the allergy discipline is a cross-cutting specialty, which transcends such boundaries, in that allergists deal with conditions involving multiple systems, often managing them jointly with colleagues from “sister specialties” such as respiratory medicine, pediatrics, dermatology, ophthalmology, otorhinolaryngology and gastroenterology. However, the terms currently used on a daily basis by allergists are not all widely known by practitioners from the “sister specialties” and their absence from health classification and coding systems contributing to the misclassification and/or under-representation of these entities in official health statistics and, as a consequence, a tremendous negative impact at many different levels on the management of allergic disorders.

#### Understanding of the underlying mechanisms

Medical science’s understanding of the immune system has advanced significantly over the last decade having positive implications for both research and clinical practice. This development has led to an increasing number of commercially available diagnostic tools as well as a number of new products for the management of allergic and hypersensitivity conditions. In general, there is now an accepted understanding of the mechanisms underlying the most important of them, enabling a comprehensive range of “stem” concepts to be incorporated into ICD-11. The “stem” concepts can then be further characterized by appending further detail (e.g. triggers, chronology and severity grading) to produce a sophisticated post-coordinated expression (e.g. Drug-induced anaphylaxis: grade III: due to benzylpenicillin).

#### From a public health perspective

Integration of a comprehensive allergic and hypersensitivity conditions classification into national and international health information systems is crucial to the identification of their health impact at both a national and a global level and to help identify service deficiencies and provide the motivation for change. Misconceptions about and under-representation of common allergic disorders in healthcare coding systems hinder clinical research and contribute to a lack of ascertainment and poor recognition of their importance for health care planning, resource allocation and reimbursement.

For these reasons, we have considered it timely to update the representation of allergic and hypersensitivity conditions within ICD [10].

## Methods

### Allergy perspective of the ICD-11 revision

The ICD is a system of classifying medical diagnoses, developed and periodically revised by the WHO, providing a common language both for health records and for epidemiological analyses of disease. The WHO started to work on ICD-11 in 2007. A Revision Steering Group (RSG) has overseen some 18 content-specific Topic Advisory Groups (TAGs), each charged with updating sections of the classification relevant to it, in collaboration where necessary with other TAGs [10]. As new proposals come forward they can be viewed and commented upon the online ICD-11 beta draft [11].

The structure of ICD-11 is more complex than that of its predecessors in that it is based on a ”Foundation” from which a suite of different classifications or “views” can be extracted, of which the principal is the core ICD-11 for Mortality and Morbidity Statistics (ICD-11 MMS), the direct replacement for ICD-10. The current plans envisage that ICD-11 MMS will be finalized in 2018. In addition to the ICD-11 MMS this there is scope for more detailed specialty classifications, which could include one for Allergic disorders. The polyhierarchical structure of the Foundation, in which an entity may have more than one hierarchical parent, permits a single condition to be represented in more than one location, as explained in more detail below.

In contrast to previous revisions, the current ICD-11 draft is housed online and can receive external proposals for consideration by the TAGs, RSG and WHO.

Realizing that the current ICD revision would offer a unique opportunity to reach a better classification, definition and standardization of allergic and hypersensitivity conditions, an international collaboration of Allergy Academies has convened an Allergy Working Group (AWG) which has worked tirelessly since 2013 on providing scientific evidence to support the need for change and on developing an improved and up-to-date classification of allergic disorders.

The actions so far have been supported and acknowledged by the WHO ICD Revision Project staff, with whom the AWG has been keeping bilateral continuous dialog, and have been documented by a series of peer-reviewed publications [7, 12–19]. The construction of the “Allergic and hypersensitivity conditions” section of the new ICD-11 chapter “Disorders of the immune system” has involved numerous steps and would not have been possible if the AWG had not undertaken constant discussions with the relevant TAGs [11].

## Results

### Classification of allergic or hypersensitivity disorders involving the respiratory tract

**Fig. 1:**
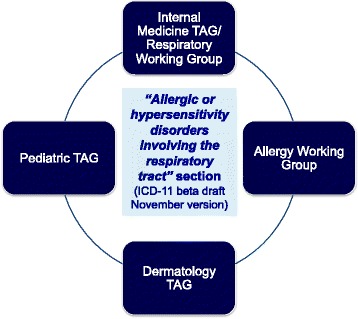
Collaboration between specialties for the construction of the “Allergic or hypersensitivity disorders involving the respiratory tract” (TAG = Topic Advisory Group)

The section of ICD-11 entitled “Allergic and hypersensitivity disorders involving the respiratory tract” has been constructed with full agreement of the involved TAGs and Expert Working Groups (EWGs) (Fig. 1). The structure was based on the overall design of the section “Allergic and hypersensitivity diseases”, which was validated by crowd-sourcing and simplified according to guidance from members of the RSG. The discussion had the scientific and technical basis to ensure comparability and consistency. An intensive exchange of e-mails, tele- and video-conferences started in February 2014 and was the basis for what was submitted into the online ICD-11 beta draft proposal platform. All the actions of the AWG have been undertaken with RSG guidance.

**Fig. 2:**
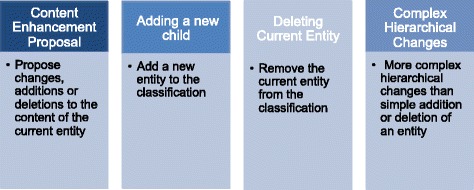
Purposes of the four main proposal type available in the ICD-11 beta draft platform

The ICD-11 beta draft supports a proposal system in which four main types of proposal are possible: i) content enhancement proposal, ii) addition of new child entity to an existing entity iii) deletion of an existing entity and iv) complex hierarchical changes (Fig. 2). Each submitted proposal has to be supported by a rationale and peer-reviewed references, and must follow an established “content model”. The ICD-11 content model incorporates: title, definition, synonyms, narrower terms, exclusions, body system, body site, signs and symptoms, causal agents and causal mechanisms [10]. The ICD-11 beta draft platform [11] can be considered a WHO web-observatory in which the members of the RSG and TAGs can monitor proposal submissions and comments and can recommend implementation or rejection of proposals.

**Fig. 3:**
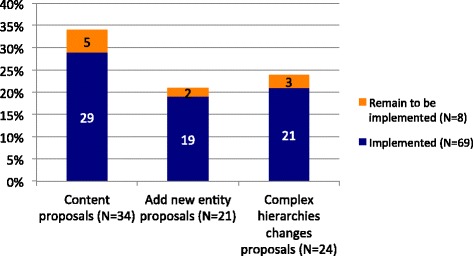
Proposals submitted into the ICD-11 beta draft platform (November 2015 version) for the construction of the “Allergic and hypersensitivity disorders involving the respiratory tract”

The AWG carried out discussions with the relevant TAGs and EWGs with whom it shared common interests; we started the submission process as soon as we reached a consensus was reached. Most of the proposals for the construction of this section were submitted between February and April 2014 but improvements have continued to be made based on comments received. During this process, the AWG submitted a total of 79 proposals, most of which (87.3%) have been implemented (Fig. 3). We did not identify any entities which required to be deleted.

**Fig. 4:**
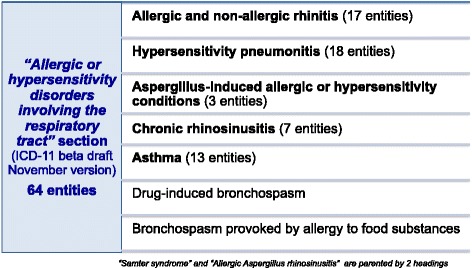
The current “Allergic and hypersensitivity disorders involving the respiratory tract” section of the ICD-11 beta draft platform (November 2015 version)

Currently, the respiratory tract allergy and hypersensitivity classification contains 64 entities distributed across 5 main categories: i) Allergic and non-allergic rhinitis, ii) Hypersensitivity pneumonitis, iii) Aspergillus-induced allergic or hypersensitivity conditions, iv) Chronic rhinosinusitis and v) Asthma. Two further entities have been added more recently: “Drug-induced bronchospasm” and “Bronchospasm provoked by allergy to food substance” (Fig. 4).

As explained above the ICD-11 Foundation is a polyhierarchical construct. Both entities Samter syndrome and Allergic Aspergillus rhinosinusitis are represented in two categories, meaning that Samter syndrome is displayed in the ICD-11 Foundation as a child both of Chronic rhinosinusitis and of Other specified asthma; and Allergic Aspergillus rhinosinusitis is displayed similarly as a child both of Aspergillus-induced allergic or hypersensitivity conditions and of Chronic rhinosinusitis. Although allergic ocular conditions, such as allergic conjunctivitis usually coexist with respiratory allergies, we did not include these disorders under the “Allergic or hypersensitivity disorders involving the respiratory tract” section since they are classified under a specific section named “Allergic or hypersensitivity disorders involving the eye”.

The ICD-11 MMS permits only one hierarchical parent for a given entity but does display entities that are located primarily elsewhere to be shown as cross-references in gray. As a consequence many of the entities to be found in the “Allergic or hypersensitivity disorders involving the respiratory tract “ section of the ICD-11 MMS are shown as cross-references from the “Diseases of the respiratory system” chapter.

## Discussion

### Lessons of the building process

The construction of a classification of respiratory allergies was a labor-intensive and complicated process. The classification forms only a part of the new section on Allergic and hypersensitivity conditions which WHO has accepted for inclusion in the new Disorders of the immune system chapter in ICD-11, thus for the first time enabling these disorders to be properly represented in ICD.

In view of the fact that “Allergy” as a specialty crosses boundaries between disciplines, many allergic and hypersensitivity conditions are also relevant to other specialties. Intensive cooperation between the representatives of different specialties enabled consensus to be reached for the construction of the “Allergic or hypersensitivity disorders involving the respiratory tract” section. As an example the proposal that “Bronchospasm provoked by allergy to food substances” should be placed within “Allergic or hypersensitivity disorders involving the respiratory tract” was put forward by the Dermatology TAG and then agreed by the Respiratory EWG of the Internal Medicine TAG, the AWG and the Pediatric TAG.

More than a mere discussion of classification, these interactions helped us to develop a common vision for handling concepts, terminology and ontology. Throughout this systematic process and after a fruitful discussion we were able to capture the “external” real understanding of how allergy and hypersensitivity conditions are perceived by others. This provided us with a global view of the key issues to be managed in order to strengthen the allergy specialty.

## Conclusion

The establishment of the ICD-11 “Allergic or hypersensitivity disorders involving the respiratory tract” section, as well as the other sessions of the “Allergic and hypersensitivity conditions” chapter will allow the dissemination of the updated concepts to be used in clinical practice by many different specialties and health professionals.

## Abbreviations

AWG: Allergy working group
CRDs: Chronic respiratory diseases
COPD: Chronic obstructive pulmonary diseases
EU: European Union
EWGs: Expert working groups
ICD: International classification of diseases
ICD-11 MMS: ICD-11 for mortality and morbidity statistics
RSG: Revision steering group
TAGs: Topic advisory groups
WHO: World Health Organization
